# Shear wave elastography findings in Immunoglobulin A Nephropathy patients: is it more specific and sensitive for interstitial fibrosis or interstitial fibrosis/tubular atrophy?

**DOI:** 10.1080/0886022X.2020.1779087

**Published:** 2020-06-28

**Authors:** Kenan Turgutalp, Yüksel Balcı, Caner Özer, Simge Bardak, İclal Gürses, Yasemin Karabulut, İlter Helvacı, Esra Dölarslan, Serap Demir, Ahmet Kıykım

**Affiliations:** a Department of Internal Medicine, School of Medicine, Division of Nephrology, Mersin University, Mersin, Turkey; b Department of Radiology, School of Medicine, Mersin University, Mersin, Turkey; c Department of Nephrology, Lefkosa BN State Hospital, Lefkosa, Cyprus; d Department of Pathology Cerrahpasa School of Medicine, Istanbul University – Cerrahpasa, Istanbul, Turkey; e Department of Pathology, School of Medicine, Mersin University, Mersin, Turkey; f Department of Business Information and Biostatistic Management, Silifke School of Applied Technology and Management, Mersin University, Mersin, Turkey

**Keywords:** Immunoglobulin A Nephropathy; shear waveelastography; interstitial fibrosis, Young’s Elastic Modulus

## Abstract

**Background:**

Prediction of prognosis in Immunoglobulin A Nephropathy (IgAN) and taking appropriate precautions may reduce annual incidence of chronic kidney disease. This may be possible by close follow-up for the development and progression of interstitial fibrosis (IF) or interstitial fibrosis/tubular atrophy (IFTA) in IgAN patients.

**Aim:**

To investigate whether Young’s elastic modulus (YM) which measured shear wave elastography (SWE) might be used for follow-up of IF or IFTA in IgAN patients.

**Methods:**

Prospective study was approved by Human Research Ethics Committee. Group 1 consisted of patients with IgAN. Group 2 consisted of healthy control participants. Young’s elastic modulus which is a value of stiffness along with longitudinal stiffness was used to evaluate tissue elasticity. Specificity, sensitivity, positive predictive value (PPV) of YM for the presence of IF and IFTA were evaluated.

**Results:**

Group 1 consisted of 30 participants, and group 2 consisted of 32 participants. Sensitivity and specificity of SWE to diagnose presence of IF for YM > 15 kPa were 89% and 90%, respectively. PPV among the ones whom IF was diagnosed by YM >15 kPa was 91%. Sensitivity and specificity of SWE to diagnose presence of IFTA for YM > 15 were 65% and 51%, respectively. PPV among the ones whom IFTA was diagnosed by YM >15 kPa was 78.1%.

**Conclusions:**

YM which measured SWE is highly specific and sensitive in the diagnosis of IF, but not for IFTA in IgAN patients. Therefore, progression for IF in IgAN may be followed by SWE.

## Introduction

Immunoglobulin A Nephropathy (IgAN) is the most common primary glomerulonephritides worldwide [1]. Primary glomerulonephritides are the third most common cause of chronic kidney disease (CKD) [2]. Prediction of prognosis in IgAN and taking appropriate precautions may reduce annual incidence of CKD. This may be possible by close follow-up for the development and progression of interstitial fibrosis (IF) or interstitial fibrosis/tubular atrophy (IFTA) in IgAN patients [3].

Interstitial fibrosis was considered in Oxford 2009 tubular classification [4], whereas evaluation of IFTA was emphasized in the Oxford 2016 classification of IgAN [3]. Degree of IF or IFTA is associated with prognosis of IgAN. Unfortunately, serum creatinine, estimated glomerular filtration rate (eGFR), and urinalysis which had been used in clinical practice are inadequate parameters for follow-up of IF or IFTA in patients with IgAN. Despite all the disadvantages, eGFR is still the best predictor of IF or IFTA as there was no alternative method, and is an important indicator of prognosis [5].

Shear wave elastography (SWE) is a noninvasive test that may measure stiffness in the tissue. Young’s elastic modulus (YM) measured by SWE correlates with the degree of IF [6,7]. SWE may be an ideal method for long-term follow-up in IgAN as it is a noninvasive method which does not require radiocontrast agent use. This method has been used to demonstrate fibrosis in patient with liver cirrhosis. Using SWE, fibrosis can be assessed also in patients without cirrhosis, such as breast, thyroid, prostate diseases, and renal allograft [8]. There is not enough knowledge about whether SWE can be used for the monitoring of IF and IFTA in patients with IgAN.

The aim of this study was to investigate whether YM which measured SWE might be used for follow-up of IF or IFTA in IgAN patients. We tried to determine which of the Oxford classification is related more closely with SWE.

## Methods

### Subjects

Prospective study design was approved by Human Research Ethics Committee (09/02/2017, 2017/29). Study was performed between March 2017 and September 2018. Patients were followed as outpatients by Mersin University School of Medicine, Nephrology department. Informed consent was obtained from patients.

Group 1 consisted of patients who had diagnosis of IgAN by renal biopsy. Group 2 consisted of healthy control participants who did not have any comorbidities, urinary sediment abnormality, or abnormal biochemical test results.

### Exclusion criteria

For group 1, patients aged below 18 years, pregnant patients, renal transplant recipients, patients with diabetic kidney disease, patients with a body mass index > 35 kg/m^2^, congestive heart failure (CHF), acquired or congenital renal cysts, polycystic kidney disease, medullary cystic disease, medullary sponge kidney, renal vein thrombosis, nephrolithiasis, hydronephrosis, renal artery stenosis, renal mass, chronic obstructive pulmonary disease (COPD), Alzheimer’s disease, active infection, cerebrovascular disease (CVD), malignancy, cirrhosis, Parkinson’s disease, adrenal insufficiency, acute kidney injury, retroperitoneal hemorrhage, subcapsular hematoma, or intraparenchymal hemorrhage after renal biopsy and patients who declined to participate in the study were all excluded. Patients who had renal biopsy more than 1 week ago, and patients who initiated treatment within 1 week, secondary glomerular diseases, primary glomerular diseases other than IgAN, secondary IgAN, and crescentic IgAN were also excluded.

For group 2, patients aged below 18 years, patients with comorbidities, urinary sediment abnormalities, or biochemical abnormalities were excluded.

### Imaging data

SWE was performed for group 1 patients within seven days after the renal biopsy before initiation of treatment. In addition to this, SWE was also performed for group 2 patients. The SWE examinations were performed by radiologist with more than 15 years of experience, who was blinded for the patient clinic data and renal biopsy results. Elastography results were recorded to electronic medical recording system just after the procedure. Ultrasonography (US) and SWE examinations were carried out with low-frequency (3.5–5 MHz) convex transducer, Canon Medical System (Otawara, Japan).

First, longitudinal dimensions of kidneys were measured in supine position with B mode. SWE evaluation was performed. SWE was used for the evaluation of kidney elasticity. YM which is a value of stiffness along with longitudinal stiffness was used to evaluate tissue elasticity. The tissue stiffness is quantified with YM, defined by the ratio between the applied stress and the induced strain and expressed in kPa [9].

US pulses were applied to the target tissue for a very short time (0.003–0.4 ms) and acoustic radiation force was exerted. During SWE imaging, patients in lateral decubitus position were told to hold their breath. The probe was held in the sagittal plane, close to the kidney. A rectangular area (15 × 10 mm) within the renal cortex was assigned as the region of interest (ROI). ROI was set next to the inferior pole of the cortex to exclude vessels and to obtain the best acoustic window. Minimal pressure was applied on the probe. Distance between ROI and skin was noted. Five measurements were performed for each patient and average was taken. Measurements were performed from right kidney due to its more appropriate depth. Measurements were performed from left kidney when the right kidney was located deeper or a suitable acoustic window could not be obtained.

For renal USG evaluation, longitudinal dimension between 10 and 12 cm and renal parenchymal thickness between 20 and 30 mm were accepted as normal [10].

### Pathologic data

Renal biopsies were interpreted by a pathologist with more than 25 years professional experience (İ.G.).

Presence and grade of TA in renal biopsy specimens of group 1 patients were evaluated according to the Oxford classification of IgAN 2009 [4]. TA was defined by the presence of thick irregular tubular basement membranes with reduced tubular diameter. It was scored according to the percentage of cortical area involvement, <1% was denoted as absence of TA, 1–5% rounded to 5% (moderate TA), and other values rounded to the nearest 10% (severe TA) [4]. IF was defined as increased extracellular matrix separating tubules in the cortical area. It is scored as percentage involvement, <1% was denoted as absence of IF (stage F_0_), with 1–5% rounded to 5% (stage F_1_, moderate IF), and other values rounded to the nearest 10% (stage F_2_, severe IF) [4]. The percentage of the cortical area involved by tubular atrophy or IF was quantitated [3]. Grading of IFTA was performed according to Oxford classification of IgAN 2016. If percentage of IFTA ≤25%; stage T_0_ (mild IFTA), 26–50%; stage T_1_ (moderate IFTA), >50%; stage T_2_ (severe IFTA) [3].

Cutoff values of YM for IF and IFTA were obtained.

### Demographic and laboratory data

Laboratory data, demographic and ultrasonographic features of two groups were compared and associations were evaluated.

Arterial pulse, systolic blood pressure (SBP), and diastolic blood pressure (DBP) of all participants were measured. Blood samples were taken after 8 h of fasting for hemoglobin, serum glucose, blood urea nitrogen (BUN), and creatinine.

Twenty-four hour urine was collected to determine 24-h protein excretion. It was performed twice and average was calculated. eGFR was calculated by CKD-EPI formula (Chronic Kidney Disease Epidemiology Collaboration) = 141 × min(Scr/κ,1)*α*×max(Scr/κ,1) – 1.209 × 0.993age × 1.018 [female] × 1.159 [black]) [11].

Serum plasma glucose, BUN, and creatinine were measured by Olympus AU 640 Chemistry Immunoanalyzer (Tokyo, Japan).

Presence of comorbidities like CHF, hypertension (HT), diabetes mellitus (DM), COPD, Alzheimer’s disease, CVD, malignancy, CVO, cirrhosis, Parkinson’s disease, and adrenal insufficiency were evaluated.

### Associated factors with YM

Association between IFTA stages (stages T_0_, T_1_, and T_2_) and YM was investigated in group 1. Association between IF stages (stages F_0_, F_1_, and F_2_) and YM was also evaluated in group 1. Light microscopic images of renal biopsy specimens were obtained. IF and IFTA stages in light microscopic examination were compared with YM.

- YM of group 2 was measured.
- YM of group 1 and group 2 was compared.

Associations between YM and 24-h urine protein excretion, ultrasonographic renal dimensions (URDs), gender, age, eGFR, ultrasonographic renal parenchymal thickness (URPT), and serum creatinine were also investigated.

Possible influencing factors for YM were investigated.

Specificity, sensitivity, accuracy, positive predictive value (PPV), and negative predictive value (NPV) for the presence of IF and IFTA were evaluated.

### Associated factors with IF and IFTA

Relation between IF and 24-h urine protein excretion, URD, gender, age, eGFR, URPT, and serum creatinine were investigated. Relation between IFTA and 24-h urine protein excretion, URD, gender, age, eGFR, URPT, and serum creatinine were also investigated.

Specificity, sensitivity, accuracy, PPV, and NPV of eGFR for the presence of IF and IFTA were evaluated.

### Associated factors with URD and URPT

Specificity, sensitivity, accuracy, PPV, and NPV of both URD and URPT for the presence of IF and IFTA were evaluated.

### Statistical analysis

MedCalc packet program was used for statistical analysis. The mean ± standard deviation was used for descriptive statistics for non-normally distributed variables. For the mean comparisons of the groups, where more than two groups involved, one-way analysis of variance was applied. ROC curve analysis was used to evaluate diagnostic performance of YM and eGFR for IF. Area under the ROC curve was calculated. Besides sensitivity, specificity, PPV and NPV, accuracy values were calculated.

YMs were converted to three level ordinal categorical level (percentile and cutoff methods) in order to analyze associations between YM and IFTA (stages T_0_, T_1_, and T_2_) and IF (stages F_0_, F_1_, and F_2_) according to the Oxford classification of IgAN 2016 and 2009, and Kendall’s Tau-b statistic was used for compliance analysis.

Pearson’s correlation analysis was used to test linear association between eGFR and YM and scatter plot was performed. Ordinal logistic regression analysis was used to evaluate the importance of each variable (such as age, gender, eGFR, ROI, IF, and IFTA) in determining YM.

## Results

Group 1 consisted of 30 participants (15 males, 15 females), and group 2 consisted of 32 participants (16 males, 16 females). Mean age was 40.2 ± 11.3 years for group 1 and 39.2 ± 8.6 years for group 2 (*p*> .05).

In group 1, distribution of participants according to IF was as follows: stage F_0_: *n* = 9, F_1_; *n* = 10, F_2_; *n* = 11, whereas distribution of participants according to IFTA was as follows: stage T_0_: *n* = 9, T_1_; *n* = 11, T_2_; *n* = 10 patients.

There were statistically significant difference between serum creatinine, YM, 24-h urine protein excretion, parenchymal thickness, and eGFR values of groups 1 and 2 (*p*<.05), whereas there was no difference between ROI and kidney dimensions of groups 1 and 2 (*p*>.05).

*Cutoff values of YM*: In case of absent IF and mild IFTA, YM was between 0 and 15 kPa, whereas YM was 16–27 kPa for moderate IF and moderate IFTA, and >28 kPa for severe IF and severe IFTA. Laboratory data, demographic and ultrasonographic features of groups are shown in Table 1.

**Table 1.** Laboratory data, demographic and ultrasonographic features of all patients (mean values).

	Group 1 (*n* = 30)	Group 2 (*n* = 32)	*p* Value
Systolic BP (mmHg)	132.5 ± 12.5	120.7 ± 0.7	*<.05*
Diastolic BP (mmHg)	83.5 ± 7.7	81.2 ± 0.9	>.05
Pulse rate	84.3 ± 4.7	85.9 ± 6.1	>.05
Urea (mg/dL)	34.2 ± 2.1	25.2 ± 3.1	*<.05*
Creatinine (mg/dL)	1.71 ± 0.8	0.6 ± 0.1	*<.05*
FBG (mg/dL)	96.4 ± 30.1	92.2 ± 4.2	>.05
Proteinuria (mg/day)	1813.5 ± 1390.5	80.2 ± 21.5	<.05
eGFR (mL/minute)	58.9 ± 34.3	118.5 ± 4.9	<.05
URD (mm)	103.4 ± 15.1	112.3 ± 10.9	*>.05*
URPT (mm)	14.5 ± 3.1	22.6 ± 2.3	<.05
ROI skin distance (mm)	36.3 ± 12.1	37.2 ± 4.3	*>.05*
YM (kPa)	25.9 ± 13.6	9.9 ± 2.8	<.05

Italic values represents as significance values. BP: blood pressure; FBG: fasting blood glucose; eGFR: estimated glomerular filtration rate; URD: ultrasonographic renal dimension; URPT: ultrasonographic renal parenchymal thickness; mm: millimeter; ROI: region of interest; YM: young module.

*Factors affecting the YM*: The factors affecting the YM are shown in Table 2. eGFR (estimated: −0.066; 95% CI: −0.094 to −0.038; *p*<.001) and IF (estimated: 0.058; 95% CI: 0.041–0.085; *p*<.001) were found to be the factors affecting the YM.

**Table 2.** Factors affecting YM.

	Estimate	Std. error	Wald	df	*p* Value	95% confidence interval	
						Lower Bound	Upper Bound
Threshold	–1.838	2.107	0.761	1	.383	–5.968	2.291
	1.046	2.068	0.256	1	.613	–3.008	5.099
Location							
Age	0.037	0.031	0.385	1	<.05	–0.025	0.098
eGFR	–0.066	0.014	20.706	1	<.05	–0.094	–0.038
ROI (mm)	0.013	0.031	0.178	1	.673	–0.048	0.074
Gender	0.921	0.816	1.274	1	.259	–0.678	2.521
IF	0.058	0.011	15.836	1	<.05	0.041	0.085
IFTA	0.031	0.026	0.379	1	.235	2.342	5.283

eGFR: estimated glomerular filtration rate; IF: interstitial fibrosis; IFTA: interstitial fibrosis/tubular atrophy; ROI: region of interest.

Multivariate ordinal logistic regression analysis to evaluate the importance of each variable in determining YM.

Association between YM and IF: YM values of stages F0, F1, and F2 in group 1 were 8.2 ± 1.3 kPa, 19.3 ± 2.4 kPa, and 47.9 ± 3.6 kPa, respectively. Chi square analysis revealed significant difference between YM and F0, F1, and F2 stages (Pearson’s Chi square *p*<.001). Kendall’s Tau-b statistics revealed a difference of 0.519 which was statistically significant between YM and F0, F1, and F2 stages (*p*<.001).

Association between YM and IFTA: YM of T0, T1, and T2 stages in group 1 were 14.4 ± 1.7 kPa, 23.3 ± 2.9 kPa, and 32.9 ± 2.6 kPa, respectively. Chi square analysis revealed significant difference between YM and T0, T1, and T2 stages (Pearson’s Chi square *p*<.026). However, Kendall’s Tau-b statistics revealed a difference of 0.185 which was not statistically significant between YM and T0, T1, and T2 stages (*p* = .332).

There was statistically significant difference between YM of stage F0 and T0, F1 and T1, F2 and T2 (for all, *p*<.05).

*Other factors associated with YM*: Significant negative correlation was present between YM and URD and URPT (*p*< .05, *r*= −0.789, *r*= −0.705 for all). Besides, significant negative correlation was present between YM and eGFR (*p*<* .05*, *r*= −0.710). There was positive correlation between YM and creatinine, age (*p*<* .05* for all, *r* = 0.415, *r* = 0.538). There was not statistical association between YM and 24-h urine protein excretion, and gender (*p*>* .05* for all, *r* = 0.181, *r* = 0.158).

**Figure 1.**
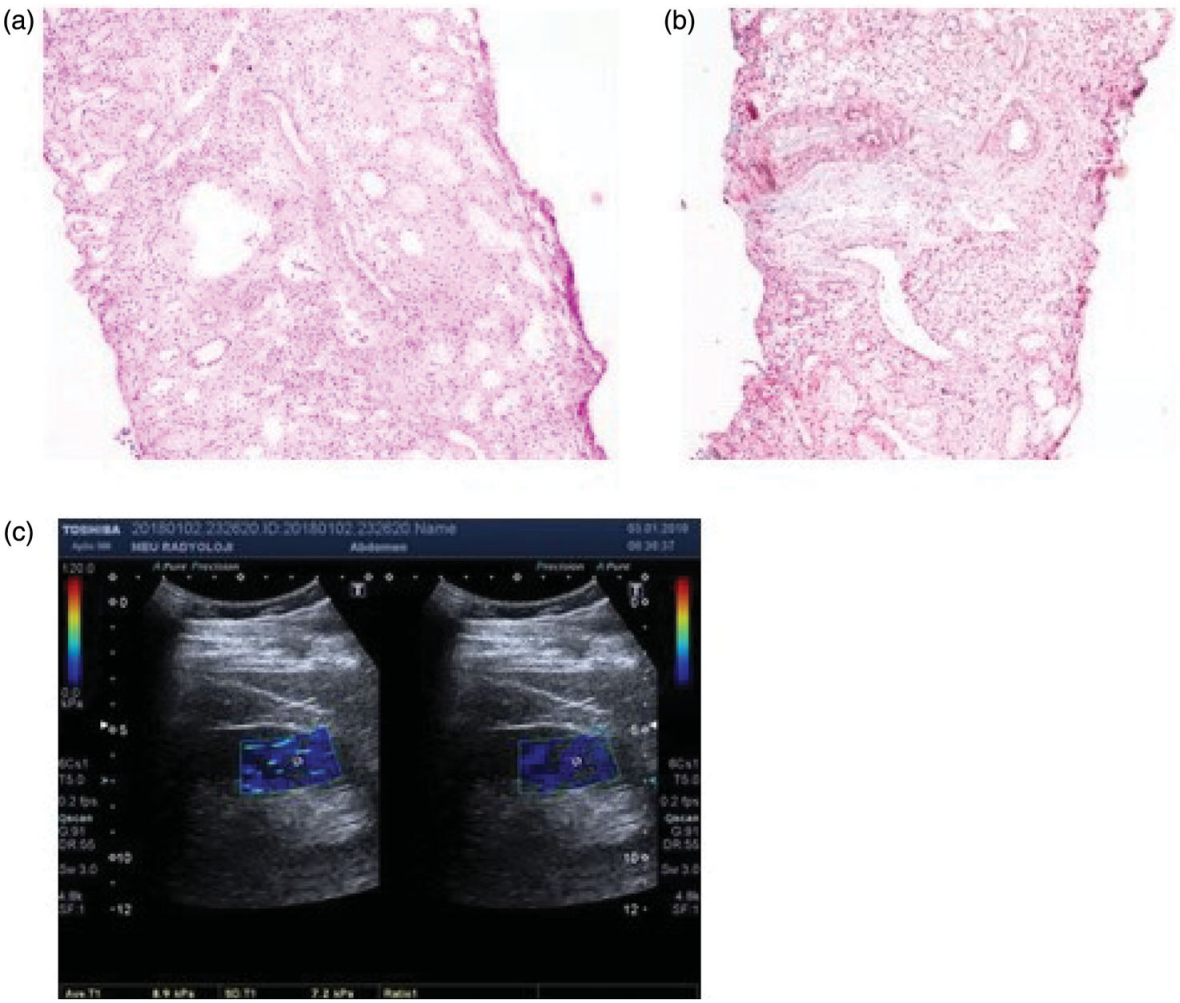
Patient 1: light microscopic images of renal tissue that stage T1 (moderate IFTA)-stage F0 (absence of IF) and their SWE image. (a) H&E ×200. (b) Masson’s trichrome ×200. (c) YM = 7.2 kPa (compatible with the stage F0 (absence of IF)).

**Figure 2.**
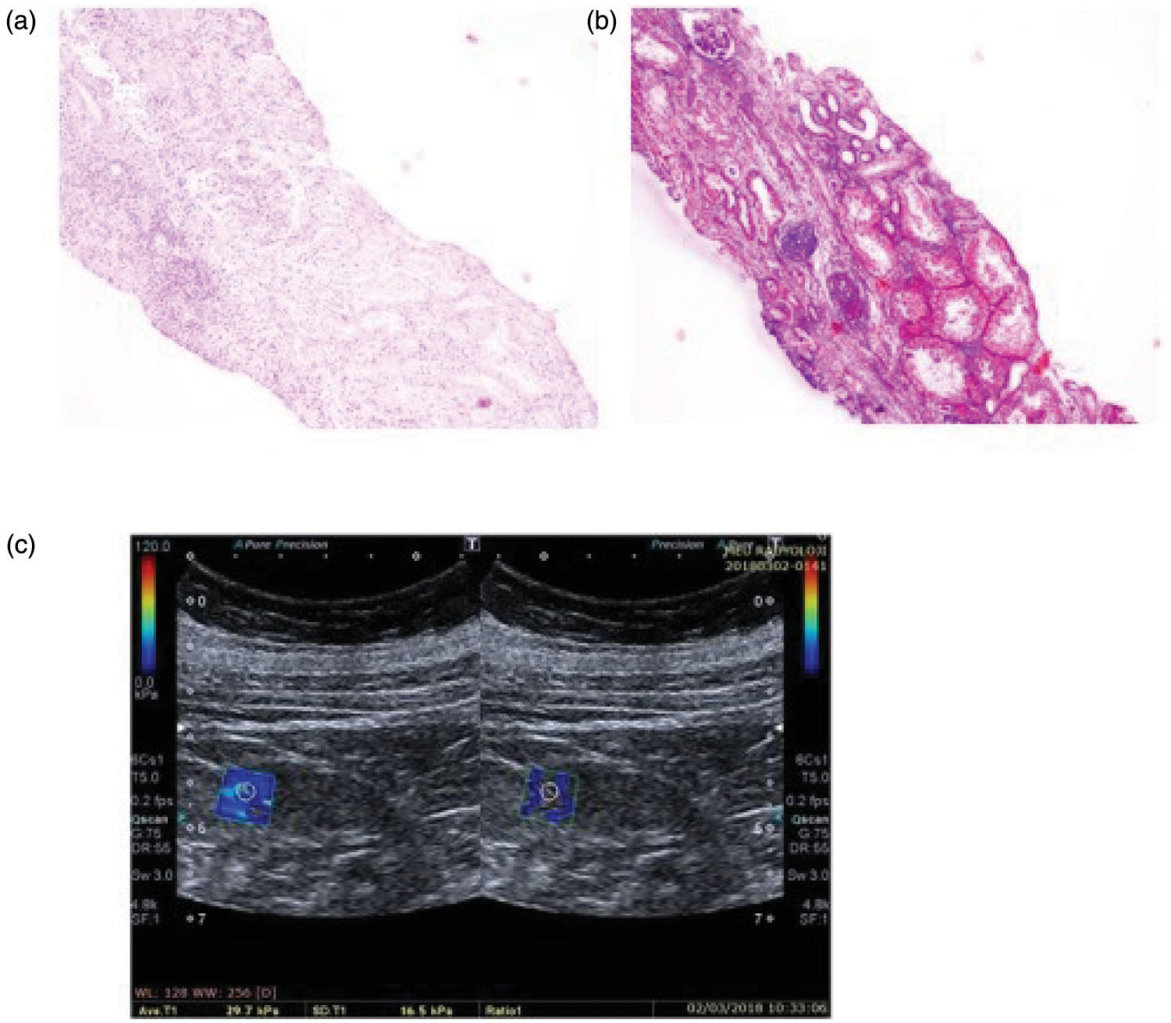
Patient 2: light microscopic images of renal tissue that stage T1 (moderate IFTA)-stage F2 (severe IF) and their SWE image. (a) H&E ×200. (b) Masson’s trichrome ×200. (c) YM = 29.7 kPa (compatible with the stage F2 (severe IF)).

**Figure 3.**
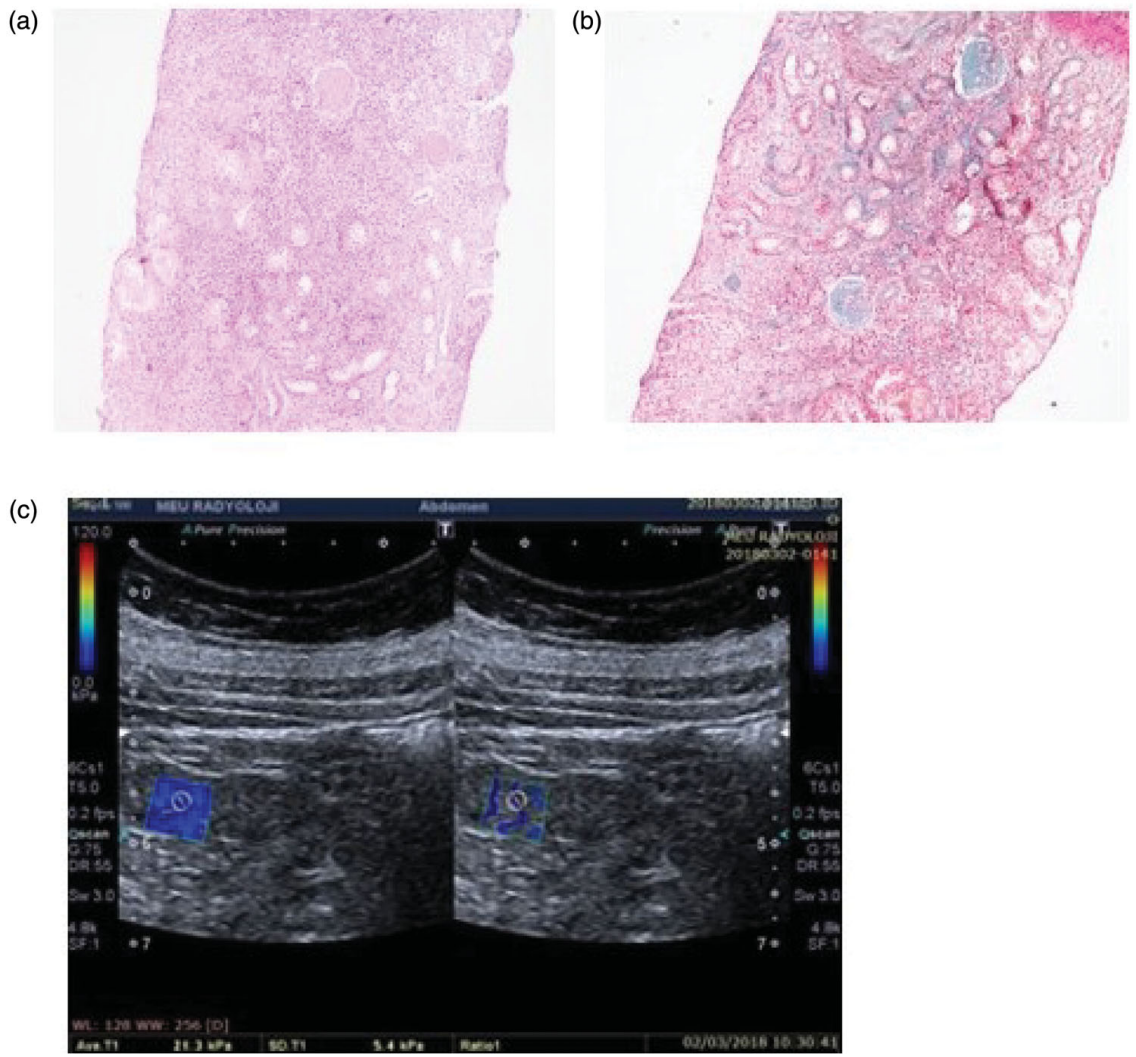
Patient 3: light microscopic images of renal tissue that stage T2 (severe IFTA)-stage F1 (moderate IF) and their SWE image. (a) H&E ×200. (b) Masson’s trichrome ×200. (c) YM = 21.3 kPa (compatible with the stage F1 (moderate IF)).

*Light microscopic findings of renal tissue and their YM images*: Light microscopic findings of renal tissue demonstrating IFTA and IF in IgAN patients and their YM images are shown in Figures 1–3. Patient 1 was found to have stage F_0_ according to the 2009 Oxford classification, but stage T_1_ according to the 2016 Oxford classification. The YM of this patient was found to be 7.2 kPa (Figure 1(a–c)). This result was consistent with stage F_0_ in Oxford 2009 classification.

Patient 2 was found to have stage F_2_ according to the 2009 Oxford classification, but stage T_1_ according to the 2016 Oxford classification. The YM of this patient was found to be 29.7 kPa. This result was consistent with stage F_2_ in Oxford 2009 classification (Figure 2(a–c)).

Patient 3 was found to have stage F_1_ according to the 2009 Oxford classification, but stage T_2_ according to the 2016 Oxford classification. The YM of this patient was found to be 21.3 kPa. This result was consistent with stage F_1_ in Oxford 2009 classification (Figure 3(a–c)).

Sensitivity, specificity, PPV, NPV, accuracy of SWE, URD, URPT, and eGFR to diagnose presence of IF are shown in Table 3. Sensitivity and specificity of SWE to diagnose presence of IF for YM > 15 kPa were found high. Similarly, PPV, NPV, and accuracy among the ones whom IF was diagnosed by YM > 15 kPa were found high. However, sensitivity, NPV and accuracy of URD and URPT for detection of IF were found to be low. Sensitivity, specificity, PPV, NPV, and accuracy of eGFR to diagnose for presence of IF were found to be high. The difference between ROC curves of SWE and eGFR (0.0316) was not statistically significant (*p*=.237).

**Table 3.** Sensitivity, specificity, PPV, NPV, accuracy of SWE, URD, URPT, and eGFR to diagnose presence of IF.

	Sensitivity	Specificity	PPV	NPV	Accuracy
SWE	89% (95% CI, 86.3–100)	90% (95 % CI, 82.4–100)	91% (95% CI, 86.3–100)	92% (95% CI, 82.4–100)	93% (95% CI, 85.2–100)
URD	43% (95% CI, 81.2–100)	84.8% (95% CI, 82.3–100)	88.5% (95% CI, 85.6–100)	42% (95% CI, 84.1–100)	57% (95% CI, 81–100)
URPT	52% (95% CI, 81.2–100)	85.2% (95% CI, 82.3–100)	86.7% (95% CI, 85.6–100)	56% (95% CI, 84.1–100)	77% (95% CI, 86.1–100)
eGFR	82% (95% CI, 85.4–100)	92% (95% CI, 82.1–100)	92% (95% CI, 85.4–100)	83% (95% CI, 81.4–100)	94% (95% CI, 89.2–100)

PPV: positive predictive value; NPV: negative predictive value; CI: confidence interval; SWE: shear wave elastography; URD: ultrasonographic renal dimensions; URPT: ultrasonographic renal parenchymal thickness; eGFR: estimated glomerular filtration rate.

Sensitivity, specificity, PPV, NPV, accuracy of SWE, URD, URPT, and eGFR to diagnose presence of IFTA are shown in Table 4. Sensitivity and specificity of SWE to diagnose presence of IFTA for YM > 15 were found low. Similarly, PPV, NPV, and accuracy among the ones whom IFTA was diagnosed by YM > 15 kPa were found low. Sensitivity and specificity of URD and URPT for detection of IFTA were low. Furthermore, PPV, NPV and accuracy of URD and URPT to diagnose IFTA were all low.

**Table 4.** Sensitivity, specificity, PPV, NPV, accuracy of SWE, URD, URPT, and eGFR to diagnose presence of IFTA.

	Sensitivity	Specificity	PPV	NPV	Accuracy
SWE	65% (95% CI, 86.3–100)	51% (95% CI, 82.4–100)	78.1% (95% CI, 86.3–100)	49% (95% CI, 82.4–100)	54% (95% CI, 85.2–100)
URD	32% (95% CI, 83.5–100)	54.8% (95% CI, 86.3–100)	67.5% (95% CI, 80.7–100)	32% (95% CI, 81.1–100)	77.8 (95% CI, 82.1–100)
URPT	52% (95% CI, 82.2–100)	85.2% (95% CI, 84.3–100)	56.7% (95% CI, 80.7–100)	46% (95% CI, 84.1–100)	67% (95% CI, 86.1–100)
eGFR	66% (95% CI, 81.2–100)	84.6% (95% CI, 82.3–100)	68.2% (95% CI, 81.2–100)	55% (95% CI, 80.1–100)	68% (95% CI, 85.1–100)

PPV: positive predictive value; NPV: negative predictive value; CI: confidence interval; SWE: shear wave elastography; URD: ultrasonographic renal dimensions; URPT: ultrasonographic renal parenchymal thickness; eGFR: estimated glomerular filtration rate.

*Associated factors with URD and URPT*: There was significant negative association between URD and serum creatinine, age (*p*<.05, *r*= −0.445, *r*= −0.443), whereas significant positive correlation was present between URD and eGFR (*p*<.05, *r* = 0.460).

There was significant negative correlation between URPT and serum creatinine, age (*p*<.05, *r*= −0.443, *r*= −0.442) whereas significant positive correlation was present between URPT and eGFR (*p*<.05, *r* = 0.460).

*Other factors associated with IF*: There was no significant association between IF and 24-h urine protein excretion, gender (*p*>* .05* for all, *r* = 0.121, *r* = 0.099, respectively). There was significant negative association between IF and eGFR, URD, and URPT (*p*<* .05* for all, *r*= −0.811, *r*= −0.647, *r*= −0.587, respectively). Positive correlation was present between IF and serum creatinine, age, YM (*p*<* .05*, for all, *r* = 0.785, *r* = 0.675, *r* = 0.712, respectively).

*Other factors associated with IFTA*: There was no significant association between IFTA and 24-h urine protein excretion, gender, URD and URPT, YM (*p>.05* for all, *r* = 0.112, *r* = 0.215, *r*= −0.109, *r*= −0.117, *r* = 0.111).

Positive correlation was present between IFTA and serum creatinine, age (*p<.05* for all, *r* = 0.659, *r* = 0.562). There was significant negative association between IFTA and eGFR (*p<.05*, *r*= −0.791).

## Discussion

The association between YM and IF was investigated in many trials in CKD and renal transplantation, but not in IgAN. To the best of our knowledge, our study was first prospective controlled study which investigated specificity and sensitivity of YM in determining IF and IFTA. Besides, correlation between YM and Oxford classification was found out.

In this prospective study, sensitivity, specificity, NPV and PPV of SWE for IF in IgAN were found quite high but SWE was not specific and sensitive for IFTA. Significant correlation was found between YM and pathological classification according to IF, whereas there was no correlation when pathological classification was performed based on IFTA. In addition, in this study, the IF in IgAN patients was found to be an influencing factor for YM. IgAN is a nephritis with IF and IFTA [12]. Both IF and IFTA are factors that determine renal survival in IgAN patients. Noninvasive methods are required for the follow-up of renal survival. Long-term follow-up of IF or IFTA in IgAN is important [12,13]. SWE is a simple and noninvasive method for the assessment of renal tissue elasticity. In comparison to the other types of US technologies, SWE is more specific and sensitive for fibrosis [14]. Although specificity of SWE for fibrosis in liver cirrhosis, breast, and thyroid diseases was proved [15–21], studies in kidney diseases were still inadequate. Trials investigating specificity and sensitivity of SWE for renal fibrosis were mostly performed in renal transplant and CKD patients and significant results were reported [14,22–29]. However, it was unclear whether follow-up of IF and IFTA may be performed by SWE. Nakao et al. compared YM and biopsy findings of 27 renal transplant patients. They found that elasticity score was significantly associated with IF and eGFR. Significant difference was found between YM of patients with eGFR > 50 mL/min and <50 mL/min [29]. SWE was not associated with IF in some trials performed in native kidneys. However, limited number of patients, preliminary design, and absence of healthy control group were limitations of these studies [30,31]. Guo et al. found strong correlation between renal histologic score and YM. In this trial, they emphasized that SWE may take place of renal biopsy for the determination of IF [27]. Samir et al. found that SWE in CKD was significantly different from healthy control. However, they did not compare histopathological IF score and SWE [32]. In our study, correlation was found between severity of IF and YM in IgAN patients who had IF in biopsy. In addition to this, YM was significantly higher in IgAN patients with IF than control group. Peng et al. carried out a study in patients with IgAN, minimal change disease, focal segmental glomerulosclerosis, membranous nephropathy and CKD, and found an association between YM and severity of IF, TA [33]. IgAN patients were classified according to Oxford Classification M_0_ (M = mesangial sclerosis), M_1_, E_0_ (E = endocapillary sclerosis), E_1_, T_0_ (T = tubular atrophy/interstitial fibrosis), T_1_ and patients with all stages were included into the study. Peng et al. found that the TA and YM were compatible. This result was not consistent with the outcome of our study. SWE may not be specific for mesangial sclerosis, endocapillary proliferation, and TA. This might confound statistical results in Peng’s study. Also, there was no healthy control group in Peng’s study. In that study, characteristics of patients with IgAN and exclusion criteria were not well defined [33]. In addition, in our study, IF was a factor that affects the YM. In contrast, IFTA was not a factor affecting the YM. This is because; IF is associated with tissue stiffness but not IFTA. In our study, we found that YM was specific and sensitive when IF alone was considered in IgAN, but not specific when classified according to IFTA. For this reason, IF may be followed by SWE in IgAN.

Increase in proteinuria in the initial phase of IgAN was associated with severity of the disease [34]. Persistent proteinuria may lead to tubular inflammation, TA, IF, and increased intraglomerular pressure, which all may lead to irreversible injury [35]. Proteinuria may decrease by the development of glomerulosclerosis [36]. Therefore, amount of proteinuria may not be associated with the IF or IFTA. Lin et al. reported significant correlation between 24-h urine protein excretion and YM in CKD [37]. They found that YM increases as CKD stage progresses. As there was a significant association between 24-h urine protein excretion and YM, they supposed that there may be an association between 24-h urine protein excretion and fibrosis. However, they did not perform biopsy in their trial, and they did not compare directly histopathologic score and 24-h urine protein excretion. Goya et al. found significant association between YM and proteinuria in diabetic nephropathy [38]. Goya et al. grouped patients according to 24-h urine protein excretion without considering histopathologic scoring and compared YM. This is far from comparing IF and 24-h urinary protein excretion. In many trials, 24-h urine protein excretion was considered as prognostic factor. However, upper threshold indicating poor prognosis is unknown [38]. Chen et al. pointed out that daily urine protein excretion above 500 mg indicated poor prognosis in IgAN [34]. In another trial, they emphasized that threshold was 1 g [39]. Therefore, 24-h urine protein excretion was insufficient to predict progression of fibrosis in IgAN [40,41]. We need markers other than 24-h urine protein excretion which may help us to predict presence and progression of IF or IFTA. In our trial, although we found association between IF and YM, no association was documented between YM and 24-h urine protein excretion. In our study, no association was found between IF, IFTA and 24-h urine protein excretion. According to this result, SWE may be more useful than 24-h urine protein excretion for the follow-up of IF in IgAN.

In adults, as age passes eGFR decreases and histopathologic findings like IF, TA occur [42]. In one study, there was no association between YM and age [43]. Similarly, Goya et al. carried out a study in diabetic nephropathy patients and healthy control group and reported that YM was significantly associated with serum creatinine, and eGFR whereas YM was not found associated with age [38]. In our study, age was found to be associated with IF, IFTA, YM, URD, and URPT. In CKD, age should be considered when SWE is performed. However, in another study performed in 45 patients with CKD, no association was found between eGFR and YM [38]. Small number of patients was the limitation of the study. Increase in serum creatinine and decrease in eGFR may be related to the progression of IF [27]. However, serum creatinine may be affected from extrarenal factors such as gender, age, race, use of trimethoprim, cimetidine, or fibric acid [44]. Despite the handicaps of eGFR in determination of IF, there was no alternative method in clinical practice. Although we found high sensitivity and specificity of eGFR for the determination of IF, we know that serum creatinine may be affected from various factors like the drugs used. New methods are needed to show the progression of IF in addition to serum creatinine and eGFR.

In our study, we found that IF, IFTA, and YM were significantly associated with eGFR and serum creatinine. In our study, there was no difference between eGFR and SWE. SWE may be an adjunct method to eGFR to diagnose IF. Low eGFR and YM > 15 kPa measured by SWE in patients with IgAN indicated presence of IF. Early diagnosis and timely intervention of IF may slow the progression and improve quality of life of the patients with IgAN.

Small kidney URD and thin URPT were reported as indicators of glomerular sclerosis and IF in some trials [45,46]. However, these were late signs of IF [47]. SWE may help early diagnosis of IF [23]. In our trial, URD and URPT had low sensitivity, but high specificity for the diagnosis of IF. IF cannot be excluded in patients with normal URD and URPT. URD and URPT had a high PPV and low NPV for IF. However, sensitivity, specificity, PPV and NPV values for YM to diagnose IF were high. Therefore, YM may be used for early diagnosis and follow-up of IF. In our study, correlation was also present between YM and URD and URPT. At the same time, in our study, URD and URPT sensitivity, specificity, PPV, NPV were not significant in detecting IFTA. Besides, URD and URPT were not related to IFTA. The cause of this may be presence of TA in IFTA. TA is defined as wasting of tubules as a result of ischemia, obstruction, or severe cellular injury. The tubular cells are usually reduced in size and filled with casts. The tubular basal membranes are often thickened. However, interstitial expansion by collagen-rich matrix and increased fibroblasts were present in IF definition, but not in TA [48]. URD and URPT may not be affected by this process. This result indicates the importance of SWE for identification and early diagnosis of IF.

### Limitations

One of the limitations of our study was the limited number of the patients enrolled. Subgroup analysis could not be performed. Second, YM was not measured after the treatment in IgAN patients. Third, distance between ROI and skin was <4 cm in our patients. For this reason, results were not applicable for the countries where people have distance between ROI and skin was >4 cm.

## Conclusions

YM which measured SWE is highly specific and sensitive in the diagnosis of IF, but not for IFTA in IgAN patients. In future, SWE may be used in clinical practice to follow IF in IgAN. However, these results should be confirmed by randomized controlled trials, and long-term studies with higher number of patients, including treatment periods.
